# Simultaneous clustering and variable selection: A novel algorithm and model selection procedure

**DOI:** 10.3758/s13428-022-01795-7

**Published:** 2022-09-09

**Authors:** Shuai Yuan, Kim De Roover, Katrijn Van Deun

**Affiliations:** 1 Section Leadership and Management, University of Amsterdam, Amsterdam, The Netherlands; 2 Department of Methodology and Statistics, Tilburg University, Tilburg, Netherlands

**Keywords:** Clustering, High-dimensional data, Variable selection, Model selection

## Abstract

The growing availability of high-dimensional data sets offers behavioral scientists an unprecedented opportunity to integrate the information hidden in the novel types of data (e.g., genetic data, social media data, and GPS tracks, etc.,) and thereby obtain a more detailed and comprehensive view towards their research questions. In the context of clustering, analyzing the large volume of variables could potentially result in an accurate estimation or a novel discovery of underlying subgroups. However, a unique challenge is that the high-dimensional data sets likely involve a significant amount of irrelevant variables. These irrelevant variables do not contribute to the separation of clusters and they may mask cluster partitions. The current paper addresses this challenge by introducing a new clustering algorithm, called Cardinality K-means or CKM, and by proposing a novel model selection strategy. CKM is able to perform simultaneous clustering and variable selection with high stability. In two simulation studies and an empirical demonstration with genetic data, CKM consistently outperformed competing methods in terms of recovering cluster partitions and identifying signaling variables. Meanwhile, our novel model selection strategy determines the number of clusters based on a subset of variables that are most likely to be signaling variables. Through a simulation study, this strategy was found to result in a more accurate estimation of the number of clusters compared to the conventional strategy that utilizes the full set of variables. Our proposed CKM algorithm, together with the novel model selection strategy, has been implemented in a freely accessible R package.

## Introduction

Recent technological developments have made it fairly easy to collect a large number of variables within a single study in social and behavioral sciences. Examples include examinations of genetic influences in organizational psychology (e.g., Chi et al., 2016; Arvey et al., 2016), personality psychology (e.g., Davis et al., 2019) and social psychology (e.g., Feldman et al., 2016); studies on neuroscientific foundations of behaviors in management (e.g., Waldman et al., 2019) and psychiatry research (e.g., Sun et al., 2009); research aiming to predict personality from social media footprints (e.g., Park et al., 2015); questionnaire-based studies that simply collected a comprehensive set of variables (e.g., Joel et al., 2017); as well as a combination of all these types of data (e.g., Bzdok & Meyer-Lindenberg, 2018).

A noteworthy advantage of data sets including many variables is that they provide a detailed and comprehensive view. Here, the definition of “many variables” is rather subjective and depends largely on the field of research. In behavioral sciences, one can think of data sets with more than 100 variables (Groeneveld & Rumsfeld, 2016). These types of data sets become increasingly common due to the fact that novel types of data sources are more and more often collected. Some special examples are so-called “high-dimensional” data sets where the number of variables exceeds the number of observations. In the context of cluster analysis – where the intent is to group observations in such a way that those in the same subgroup are similar to each other – using data with many variables will likely result in a more accurate estimation of subgroups and (or) a discovery of novel subgroups. In one of the very few reported attempts to cluster datasets with many variables, Mothi et al., (2019) combined clinical measures, laboratory measures, and measures derived from MRI scans of psychotic patients to form a combined data set, on which they conducted a cluster analysis and identified three sub-types of psychoses. Evidently, clustering high-dimensional data sets grants researchers an unprecedented opportunity to clarify and deepen our understanding of the heterogeneity in various social phenomena.

Although research that exploits data sets with many variables to identify subgroups is promising, it also comes with challenges. One of the most compelling challenges, as stressed by a number of scholars (e.g., Yarkoni and Westfall, 2017; Waldherr et al., 2017; Bzdok & Meyer-Lindenberg, 2018), is that these data sets may comprise a large amount of “irrelevant variables” (Fowlkes & Mallows, 1983). They are variables that do not separate clusters well and therefore do not define cluster structure. These irrelevant variables may hinder subgroup discovery by masking the cluster structure under investigation (Steinley & Brusco, 2008b). Therefore, a cluster analysis should effectively recover the cluster structure while simultaneously filtering out irrelevant variables.

The variable selection problem in cluster analysis is not a new topic and has been extensively studied since the 1980s. For example, Steinley and Brusco (2008b) have compared the performance of eight different procedures to address this problem. These approaches – most notably the Variable Selection in *K*-Means (i.e., VS-KM; Brusco & Cradit 2001), model-based variable selection (Raftery & Dean, 2006), the Clustering Objects on Subsets of Attributes (i.e., COSA; Friedman & Meulman 2004) and the relative clusterability weighting method (Steinley & Brusco, 2008a) – are well designed and have been extensively validated. However, these methods are computationally prohibitive in the presence of many variables, as the computational demand grows exponentially with the number of variables. For example, Steinley and Brusco (2008a) proposed to test all subsets of variables that pass the initial screening, where the theoretical maximum number of tests can be as high as 2^*J*^ − 1 (with *J* indicating the number of variables in the data set). Raftery and Dean (2006) and Brusco and Cradit (2001) have both proposed a forward-searching strategy that starts with an initial pair of two signaling variables and, after searching all remaining variables, adds other signaling variables one by one. This strategy, too, becomes very inefficient when there are more than 100 variables.

Other methods are available, however, that are able to simultaneously perform variable selection and clustering, with reasonable computational time for large data sets with many variables. They are, for example, Sparse *k*-means (SKM; Witten & Tibshirani 2010) and Sparse Alternate Sum (SAS; Arias-Castro & Pu 2017). Importantly, these methods have been verified in several simulation studies to entail a better performance than competing approaches, such as the aforementioned COSA (Witten & Tibshirani, 2010).

One of the important contributions of the current study is to present a novel method, which we named Cardinality *k*-means or CKM, for simultaneous variable selection and clustering (see Yamashita & Adachi 2020 for another application of the cardinality constraint on clustering). CKM essentially exploits the fact that principal component analysis (PCA) offers reasonable starting partitions to the *k*-means algorithm (hereafter called KM; Ding & He 2004; Xu et al., 2015), especially in high-dimensional data sets. Based on this connection, CKM approximates clustering solutions through sparse principal component analysis (SPCA; Shen & Huang^2008^) and, based on the initial results of SPCA, continuously updates partitions until convergence is reached. Here, the algorithm is considered to converge when all observations remain in the same cluster after another iteration of cluster updates. The “Methods” section illustrates how CKM theoretically relates to SKM and SAS, while the “Simulation studies” section reports how their performance compared.

As another important contribution, this study tackles the problem of selecting the correct number of clusters in the presence of (many) irrelevant variables. To date, despite calls to research this problem (e.g., Steinley & Brusco, 2008b, 2011), to the best of our knowledge, only Brudvig et al., (2019) has empirically addressed this issue. Brudvig et al., (2019) argued convincingly that the selection of the number of clusters is a central issue, and, perhaps more importantly, pointing out that the common practice of selecting the number of clusters using all variables may be misleading, as the irrelevant variables could mask the cluster separation, resulting in an erroneous estimation of the number of clusters. Building on Steinley and Brusco (2008a), the authors have proposed a new index to simultaneously select the number of signaling variables and the number of clusters. Unfortunately, the calculation of this index is prone to computational difficulties when dealing with data sets with a large number of variables. In the current study, we aim to expand this line of research in two ways: 1) we propose a novel strategy to select the number of clusters that might be more suitable in the presence of a large proportion of irrelevant variables and 2) within the framework of our novel strategy, we compare several methods to select the number of clusters in a simulation study. The novel strategy is based on the idea of extracting a “stable” set of variables that are deemed to be signaling variables given any number of clusters. To evaluate the novel model selection strategy we obtained the accuracy of the novel and competing model selection strategies for various clustering methods and with various test statistics.

The paper is organized as follows. We present the CKM model and the accompanying algorithm in Section “Methods”, where we also discuss the novel strategy to determine the number of clusters and several methods related to CKM. Three simulation studies are presented in Section “Simulation studies”. In the first two simulation studies, CKM is validated and compared with SKM and SAS across various conditions; while both the number of irrelevant variables and the number of clusters are treated as known information in the first simulation, only the latter is treated as known in the second. In the third simulation study, we illustrate the relative performance of the novel model selection strategy that utilizes the stable set of variables as opposed to the strategy that utilizes the full set of variables. We then proceed to illustrate the usage of CKM on a large data set that consists of over forty thousand variables in Section “Application”. Finally, in Section “General discussion”, we discuss the practical implication of CKM and the novel model selection strategy, address their limitations, and propose future research directions. to promote the method, we implemented CKM in a user-friendly *R* package “CKM” (available at https://github.com/syuanuvt/CKM).

## Methods

To develop CKM, we rely on results proven in Ding and He (2004) and Xu et al., (2015). They have shown how principal component analysis (PCA) can be used to obtain the subspace in which the clusters reside. A key advantage of this proposal, as discussed and illustrated in Xu et al., (2015), is the stability of the clusters obtained and an improved accuracy in recovering the clusters, given that the clustering process mainly operates on the reduced (i.e., low-dimensional) space. In the current paper, we develop CKM that builds upon these results in the context of sparse PCA (i.e., Shen and Huang, 2008; Adachi & Trendafilov, 2016) for effective variable selection. First, we discuss the assumed clustering model (i.e., the KM model) and how it links up to PCA. Then, we illustrate our novel idea of incorporating sparseness in a PCA-like framework to filter out irrelevant variables in the KM model. After that, we introduce an efficient algorithm designed for CKM, followed by an overview and comparison with related methods. Last, We formally introduce our novel strategy to determine the number of clusters in the presence of many irrelevant variables.

### Model specification

#### A PCA approach to solve the KM problem

Prior to our discussion of CKM, we briefly show the connection between KM and PCA. That PCA can be effectively used to find the subspace in which the clusters reside was first shown in Ding and He (2004) and later in Xu et al., (2015). Interested readers are referred to those articles for detailed derivations and proofs of the main results reported here.

For a variable-wise standardized data matrix **X** (i.e., each variable is mean-centered and re-scaled to unit variance) with *N* subjects and *J* variables (and **x**_*i*_ denotes the response vector of subject *i* where *i* ∈ 1,2,...,*N*), we assume a total number of *K* clusters to be present in the data. We define an indicator vector **c** in such a way that **c**(*i*) represents the cluster assignment of observation *i* and **c**^− 1^(*k*) comprises the indices of all *N*_*k*_ subjects in cluster *k*. The objective of KM is given in

1 $$ \begin{aligned} &\mathbf{argmin_{\mathbf{c}}}{\sum}_{k=1}^{K}{\sum}_{i \in \mathbf{c}^{-1}(k)} ||\mathbf{x}_{i} - \mathbf{m}_{k}||_{2}^{2}\\ \text{with}  &\mathbf{m}_{k}=\frac{1}{N_{k}}{\sum}_{i \in \mathbf{c}^{-1}(k)}\mathbf{x}_{i}, \end{aligned} $$

where ||.||22 refers to the squared Euclidean norm (for **x** = (*x*_1_,*x*_2_,...,*x*_*J*_), ||**x**||22 = *x*12 + *x*22 + ... + *xJ*2).

Because the optimization problem in Eq. 1 is a discrete one, typically an alternating algorithm with multiple starts is employed where each starting indicator vector is generated randomly and updated until convergence. From the multiple converged solutions, the best one is retained as the final solution; however, there is no guarantee this solution is optimal.

The major contribution of Ding and He (2004) and later Xu et al., (2015) is the proof of the equivalence between PCA and a continuous relaxation of KM and henceforth the proposal of solving KM with the help of PCA. To see this, they first introduced a partition matrix **H** (*N* × *K*) to specify the correspondence between subjects and clusters. More specifically, the element *h*_*ik*_, located at the *i*^*th*^ row and the *k*^*th*^ column of **H**, is constructed as follows,

2 $$ h_{ik} = \left\{ \begin{array}{ll} 1 & i \in \mathbf{c}^{-1}(k) \\ 0 & i \notin \mathbf{c}^{-1}(k) \end{array} \right. $$

This specification results in **H** having orthogonal columns. Moreover, **H** is directly linked with **m**_*k*_, according to

3 $$ \mathbf{m}_{k} = \frac{1}{\sqrt{N_{k}}}\mathbf{h}_{k}^{\prime}\mathbf{X}  $$

where **h**_*k*_ denotes the *k*^*th*^ column of **H**.

Combine Eqs. 3 and 1, and perform some algebraic operations (detailed in Appendix A), we arrive at

4 $$ \begin{aligned} &\mathbf{argmax_{H}}  Tr\mathbf{H}^{\prime}\mathbf{X}\mathbf{X}^{\prime}\mathbf{H} \\ s.t.  &\mathbf{H}^{\prime}\mathbf{H}=\mathbf{I}_{K}, h_{ik} \in \{\frac{0}{\sqrt{N_{k}}},\frac{1}{\sqrt{N_{k}}}\}. \end{aligned} $$

Equation 4 can be viewed as another way to formulate the objective of KM.

Instead of directly solving Eq. 4, Ding and He (2004) proposed to first address a more convenient problem by releasing the constraint that *h*_*ik*_ should be either 0 or 1*N*_*k*_. To do so, they introduced **Ĥ** as the continuous relaxation of **H** that satisfies **Ĥ** = **HR** where **R** is a rotation matrix subject to **RR**^′^ = **I**_*K*_. Also, to illustrate more explicitly the connection of Eq. 4 and PCA, **Z** **=** *X*^′^ is brought in. Then, Eq. 4 could be rephrased in

5 $$ \begin{aligned} &\mathbf{argmax_{\hat{H}}}  Tr\hat{\mathbf{H}}^{\prime}\mathbf{Z}^{\prime}\mathbf{Z}\hat{\mathbf{H}}\\ s.t.  &\hat{\mathbf{H}}^{\prime}\hat{\mathbf{H}}=\mathbf{I}_{K}, \end{aligned} $$

which is the PCA formulation yet formulated on the transposed data. A solution is attained when **Ĥ** equals the first *K* left eigenvectors of **Z**^′^**Z** that correspond to the *K* largest eigenvalues. Xu et al., (2015) proposed to estimate the partition matrix **H** from this *K*-dimensional representation of the data with a two-step approach: (1) obtain an initial partition by subjecting **Ĥ** to a multi-start KM algorithm; (2) use the partition resulting from the first step as a rational start for a KM analysis of the original data **X**.

We note that the objective in Eq. 5 can also be written as

6 $$ \begin{aligned} &\mathbf{argmin_{\hat{\mathbf{H}},\mathbf{P}}}||\mathbf{X} - \hat{\mathbf{H}}\mathbf{P}^{\prime}||_{2}^{2}\\ s.t.  &\hat{\mathbf{H}}^{\prime}\hat{\mathbf{H}}=\mathbf{I}_{K}, \end{aligned} $$

where **P** serves as the loading matrix and the expression can be seen as the least-squares formulation of PCA (for more details, the reader is referred to Guerra-Urzola et al., 2021). In Eq. 6, if the *t*^*th*^ row in **P** contains all zero elements, the *t*^*th*^ variable does not contribute to cluster separation and is therefore viewed as an irrelevant variable. Therefore, the contribution of the variables can be obtained by controlling **P**, e.g., by regularizing the variable contributions such that variables that are not associated to cluster separation are associated with only zero loadings. This forms the basis for the development of CKM, as described below.

#### A sparse PCA approach to solve KM in the presence of irrelevant variables

Let us reconsider the cluster analysis of **X** and assume that, out of all *J* variables, a total of *V* variables are irrelevant variables that do not separate clusters. The remaining (*J* − *V*) variables are therefore signaling variables. The vector **g** contains the indices of all *V* irrelevant variables, while *X*_*g*_ and *X*_−*g*_ denote the subset of the original data set that involve only the irrelevant and signaling variables, respectively. In light of Eqs. 1 and 3, we define the objective of KM in the presence of *V* irrelevant variables:

7 $$ \begin{aligned} &\mathbf{argmin_{\mathbf{c},\mathbf{g}}}(||\mathbf{X_{g}}||_{2}^{2} + {\sum}_{k=1}^{K}{\sum}_{i \in \mathbf{c}^{-1}(k)}{\sum}_{j\notin\mathbf{g}}(x_{ij}-m_{kj})^{2}\\ \text{with} & m_{kj}=\frac{1}{N_{k}}{\sum}_{i \in \mathbf{c}^{-1}(k)}x_{ij}, \end{aligned} $$

where *x*_*ij*_ and *m*_*kj*_ are the individual score of subject *i* and the mean score of cluster *k* on variable *j*, respectively. The objective represented by Eq. 7 is to minimize the total within-cluster sum of squares (also called within-SS) across all observations and variables. The first term, ||*X*_*g*_||, summarizes the within-SS over all irrelevant variables. To see this, note that a variable is considered irrelevant if its cluster-specific centroids are assumed equal; hence, these centroids are further equal to the grand mean (i.e., 0, since all variables are column-wise centered). The second term of Eq. 7 calculates the within-SS over all signaling variables. Note that **g** is added as a parameter over which Eq. 7 is optimized.

For the second part of Eq. 7, with a set of operations similar to those listed in Appendix A and B, we obtain an equivalent problem

8 $$ \begin{aligned} &\mathbf{argmax_{H, g}} Tr\mathbf{H}^{\prime}\mathbf{X_{-g}}\mathbf{X_{-g}}^{\prime}\mathbf{H}\\ s.t.  &\mathbf{H}^{\prime}\mathbf{H}=\mathbf{I}_{K}, h_{ik} \in \{\frac{0}{\sqrt{N_{k}}}\frac{1}{\sqrt{N_{k}}}\}, \end{aligned} $$

where **g** contains *V* irrelevant variables and *X*_−*g*_ denotes the subset of the original data set that only contain signaling variables. In the next section, we propose a set of procedures to determine *V*. Again, **Ĥ**, the continuous relaxation of **H**, can be used to replace **H** in Eq. 8, resulting in

9 $$ \begin{aligned} &\mathbf{argmax}_{\hat{\mathbf{H}}, \mathbf{g}} Tr\hat{\mathbf{H}}^{\prime}\mathbf{X_{-g}}\mathbf{X_{-g}}^{\prime}\hat{\mathbf{H}}\\ s.t.  &\hat{\mathbf{H}}^{\prime}\hat{\mathbf{H}}=\mathbf{I}_{K}. \end{aligned} $$

Furthermore, in the same vein as Eq. 6, Eq. 9 can be re-framed as a minimization problem. Adding the first part of Eq. 7, we obtain an optimization problem

10 $$ \begin{aligned} &\mathbf{argmin}_{\hat{\mathbf{H}},\mathbf{P}}||\mathbf{X} - \hat{\mathbf{H}}\mathbf{P}^{\prime}||_{2}^{2}\\ s.t. &\hat{\mathbf{H}}^{\prime}\hat{\mathbf{H}}=\mathbf{I_{k}}, {\sum}^{J}_{j=1}[\mathbf{row(P)}_{j} = 0]=V, \end{aligned} $$

where **row(P)**_*j*_ indicates the *j*^*th*^ row of the loading matrix **P** and [.] refers to the Iverson bracket: [*Q*] = 1 if *Q* is true and [*Q*] = 0 if *Q* is false. Equation 10 can be solved with a modification of the SPCA algorithm introduced by Adachi and Trendafilov (2016). Similar to the proposal in Xu et al., (2015), a KM analysis is then performed on **Ĥ**, resulting in an initial partition, *c*_0_, that is used for computing the final solution of the CKM analysis. Furthermore, the SPCA analysis produces an initial set of irrelevant variables **g** by selecting variables whose *K* loadings all equal zero. Subsequently, following a similar strategy as SKM and SAS, and as detailed in the next section, **c** and **g** are updated iteratively to solve Eq. 7.^1^

### Algorithm

In this section, we present the details of the algorithm for CKM with the number of clusters *K* and irrelevant variables *V* assumed to be known. The discussion on how to select *K* and *V* is deferred to Section “Model selection”. In essence, the algorithm consists of two parts. First, the sparse PCA problem defined by Eq. 10 is solved with a modified version of Unpenalized Sparse Loading PCA (USLPCA; Adachi and Trendafilov 2016). The modified version revises the structure of the imposed cardinality constraint so that the algorithm returns a selection of variables across all components (instead of per component). This optimization procedure is used because it has proven to be one of the most efficient algorithms to solve the SPCA problem with loading matrices subject to a cardinality constraint. Therefore, the result of this modified procedure is an accurate and efficient solution to the optimization problem presented in Eq. 10. From this procedure, the initial set of irrelevant variables **g**_0_ is obtained. Furthermore, the initial indicator vector **c**_0_ is obtained by performing a multi-start KM analysis on the component scores estimated from SPCA. In the second part, we solve the sparse KM problem defined in Eq. 7 by updating **c** and **g** iteratively. Both USLPCA and the sparse KM procedure are of an alternating least squares type and, in practice, they both converge to a local optimum. The full algorithm is presented in the form of pseudocode in Algorithm 1. In Appendix C, we show the derivation behind the optimization of **Ĥ**.

**Figure:**
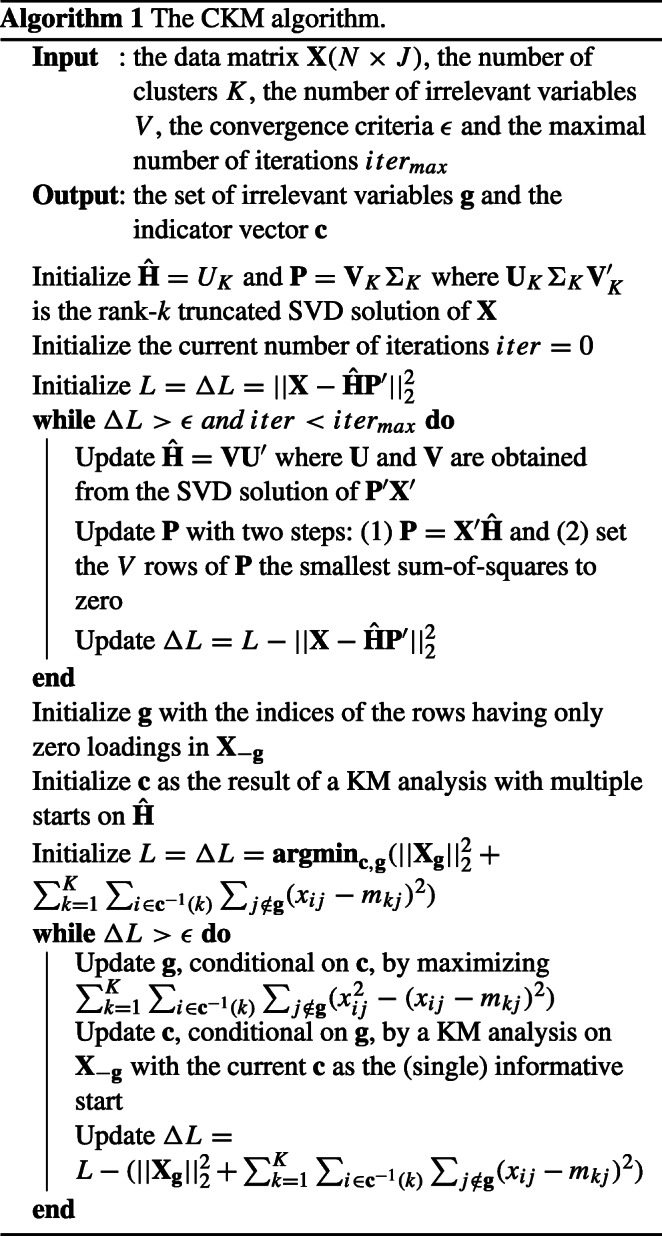

Here are four remarks on Algorithm 1. First, we solve the sparse PCA problem formulated in Eq. 10 with one rational start based on the singular value decomposition of **X**. This choice was made because this step is computationally demanding, and, in our experiments, increasing the number of starts only marginally improved the performance of the algorithm. Second, a (standard) KM analysis with ten random starts is proposed to obtain the initial cluster partition from the matrix *X*_−*g*_ with the initial set of signaling variables (i.e., **g**) obtained from SPCA. Third, if cluster recovery, but not computational efficiency, is of concern, then an additional KM analysis with ten starts can be conducted on the subset of the data set with the selected signaling variables only. The loss value from this additional analysis can then be compared to the original loss value and a final solution can be determined that minimizes this loss value. Fourth, to update the index vector of the irrelevant variables **g**, we propose to maximize $({\sum }_{k=1}^{K}{\sum }_{i \in \mathbf {c}^{-1}(k)}{\sum }_{j\notin \mathbf {g}} (x_{ij}^{2}-(x_{ij} - m_{kj})^{2})$, conditional on **c**. This can be conveniently solved by selecting the *V* variables corresponding to the *V* largest values of ${\sum }_{k=1}^{K}{\sum }_{i \in \mathbf {c}^{-1}(k)} (x_{ij}^{2}-(x_{ij} - m_{kj})^{2})$.

### Related methods

As discussed in the introduction, other algorithms that are developed from KM have been proposed to perform cluster analysis in the presence of a large number of variables. These methods could be generally classified into three types: dimension reduction, subspace clustering and variable selection. Our proposed CKM falls into the category of variable selection methods. Therefore, in the current paper, we only consider other methods from this category. Readers who might be interested in a broad review of all existing methods are referred to review articles and textbooks, for example Bouveyron and Brunet-Saumard (2014) and Bouveyron et al., (2019).

Sparse K-means (Witten and Tibshirani, 2010) was built upon the weighted *k*-means framework (Tseng, 2007) where a weight is assigned to each variable to quantify the relative importance of the variable. The objective function of SKM can be formulated in

11 $$ \begin{aligned} &\mathbf{argmax}_{\mathbf{c},w_{1}, ..., w_{J}}{\sum}_{j=1}^{J}w_{j}{\sum}_{k=1}^{K}{\sum}_{i \in \mathbf{c}^{-1}(k)} (x_{ij}^{2}-(x_{ij} - m_{kj})^{2})\\ & s.t.  w_{j} \ge 0, ||\mathbf{w}||_{2}^{2} \le 1, ||\mathbf{w}||_{1} \le s \end{aligned} $$

where *w*_*j*_ denotes the weight associated with the variable *j*, ||**w**||_1_ = ∑ *j*= 1*J*|*w*_*j*_| refers to the *l*_1_ norm, and *s* is the hyper-parameter that is determined during model tuning.

As illustrated in Eq. 11, to achieve variable selection, SKM includes a constraint with an *l*_1_ norm and a constraint with an *l*_2_ norm on the weights. The former enforces some of the weights to become exactly zero, indicating that the corresponding variables of these weights do not contribute to the clusters. The latter prevents putting all the weights on only one or a small set of variables for which the separation of the clusters is the largest. To solve Eq. 11, an alternating algorithm is developed that updates the weights and the cluster assignments iteratively. Typically, a set of equal weights is used to initialize the algorithm.

When tested on simulated data, SKM enjoyed a clear advantage over KM in terms of the accuracy of cluster recoveries, for data sets with a large proportion of irrelevant variables. However, it performed slightly worse than KM when the vast majority of variables were signaling variables.

Inspired by SKM, Arias-Castro and Pu (2017) proposed SAS, which applies a similar model as SKM, except for the fact that SAS uses binary weights *w*_*j*_: *w*_*j*_ = 1 indicates that the *j*^*th*^ variable is included in determining the cluster structure while *w*_*j*_ = 0 indicates that it is excluded. Similar to SKM, an alternating estimation procedure has been proposed that updates the weights and the cluster assignments iteratively; the authors suggested to initialize the procedure with multiple sets of randomly selected variables.^2^ In simulation studies, compared to SKM, SAS took considerably less time to achieve better performance in terms of cluster recovery in most scenarios. However, its edge over SKM in cluster recovery vanished when a vast majority of variables were irrelevant variables. We argue this is probably because the initial set of signaling variables generated is often far from the underlying model. CKM, on the other hand, uses initial values that stem from a sparse SPCA analysis of the original data; as a result, the starting set of signaling variables should be much closer to the underlying model. Therefore, we expect CKM to outperform SAS especially when the data set under consideration involves a large proportion of irrelevant variables.

### Model selection

One of our contributions in the current study is to propose a novel procedure to select *K* while taking the presence of irrelevant variables into account; in the current section, we introduce this procedure in details. Despite the fact that numerous criteria and procedures have been proposed to select *K* in deterministic clustering algorithms in general (some of the best-performing algorithms include Tibshirani et al.,, 2001 and Wang, 2010; see Steinley, 2006 for a comprehensive review), it is still largely unclear how the selection of the number of clusters should be done for these methods in the presence of irrelevant variables. In previous studies, common practice was to apply a specific criterion on the full data set, as if irrelevant variables did not influence the selection of the optimal number of clusters. We argue, however, this procedure will likely result in selecting a wrong number of clusters when a majority of variables are irrelevant and may therefore hamper an accurate recovery of the clusters. Therefore, we propose a novel strategy that filters out irrelevant variables as much as possible before selecting *K*. The procedure applies a three-step procedure, as follows. In the first step, for each possible number of clusters *K* (*K* = 1,2,...,*K*_*max*_), the optimal number of irrelevant variables *V*_*K*_ as well as the subset of signaling variables **s**_*K*_ are determined. Second, a set of variables – called the stable set or **s**_*stable*_ – are obtained that are considered as signaling variables over different values of *K*. In the third step, the optimal value of *K* (denoted by *K*_*opt*_) is determined while the associated *V*_*K*_ and **s**_*K*_ – computed during the first step – are retrieved as the optimal value of *V* and the optimal set of signaling variables, respectively.

We now first introduce the procedure to select *V*_*K*_ and **s**_*K*_ with a pre-determined value of *K*. The procedure is based on the Gap statistic (Tibshirani et al., 2001), which has demonstrated good performance in selecting the number of clusters in previous studies (e.g., Arias-Castro and Pu, 2017). More specifically, for each possible value of the number of irrelevant variables *V* (*V* = 1,2,…,*J* − 2), a CKM analysis is conducted on **X**. Note, we recommend including at least two signaling variables to avoid identification problems. From the analysis, the set of signaling variables **s**_*K*_(*V* ) is selected and its corresponding between-cluster sum of squares is calculated as *O*(*V* ). Then, *B* random data sets are generated based on the subset **s**_*K*_(*V* ) by independently permuting the observations within each variable. For each of the permuted data sets, a KM analysis is conducted, from which the between-cluster sum of squares is recorded as *O*_*b*_(*V* ). Consequently, the Gap statistic is defined in

12 $$ Gap(V) = log  O(V) - \frac{{\sum}_{b=1}^{B}log  O_{b}(V)}{B}.  $$

The intuition is that, as the permuted data contain no clusters, a larger value of *Gap*(*V* ) indicates a more salient cluster structure. Therefore, the value of *V* that maximizes *Gap*(*V* ) is selected. The corresponding set of signaling variables is consequently picked up as **s**_*K*_.

As the set of estimated irrelevant variables at each value of *K* likely differs, we identify a set of variables – the stable set of variables **s**_*stable*_ – that are consistently selected as signaling variables regardless of the value of *K*. More formally, **s**_*stable*_ is calculated as follows: **s**_*stable*_ = ∩*K*= 2*K*_*max*_**s**_*K*_, where ∩ denotes the operation of extracting the intersection over all vectors. The resulting subset of variables **s**_*stable*_ hence consists of signaling variables that were consistently identified as relevant for each and every value of *K*.

Once the stable set of signaling variables is determined, existing criteria to determine *K* can be used. Given the promising performance of the Gap statistic in recovering the true number of clusters in previous research, the Gap statistic is set as the default criterion in the implementation of our model selection procedure. However, other popular indices such as the KL index (Krzanowski & Lai, 1988) and the Dindex (Lebart et al., 1995) are interesting alternatives. In Simulation Study 3 described below, we assessed the performance of these criteria in terms of the accuracy in recovering the true number of clusters *K* across various conditions.

Last, to make the selection of *V* more precise, an additional step is recommended. This additional step determines the value of *V* from a set of candidates that are located around the selected *V* resulting from the previous step based on the Gap statistic. With respect to the size of the set of candidates, according to our experience, a set of ten alternative values is generally sufficient for the task. Specifically, the between-cluster sum-of-squares is calculated for each candidate value and an elbow point is determined to be the optimal value of *V*.^3^

A potential risk of deriving the stable set of variables in this way is that too many variables have been left out. Nevertheless, our experience in analyzing simulated and empirical data sets is that as long as *K*_*max*_ is set at a reasonable value, the identified **s**_*stable*_ always contains an adequate set of variables for selecting *K*.

Algorithm 2 summarizes the proposed model selection procedure that consists of the selection of the number of clusters *K*, and the set of signaling variables.

**Figure:**
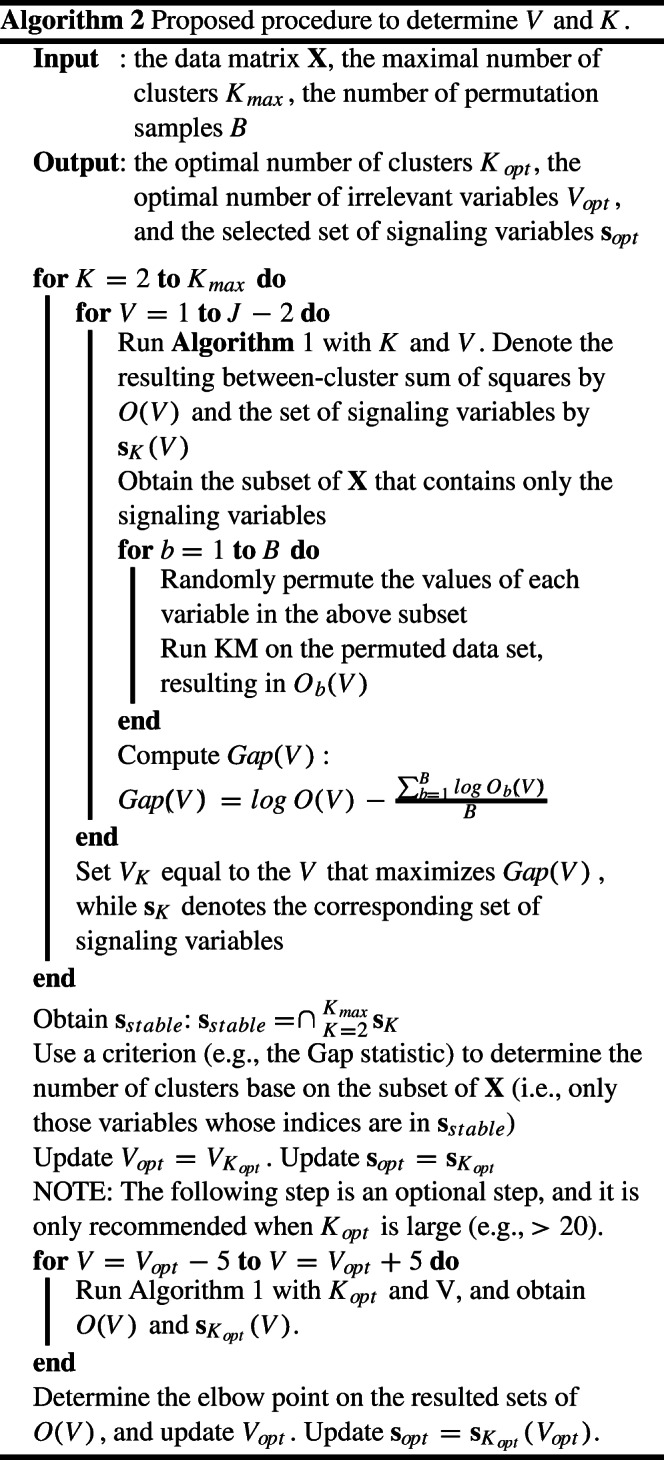

When the number of variables *J* is small, it is feasible to search the full grid (i.e., from 1 to *J* − 2) in selecting *V*_*opt*_. However, this approach is computationally prohibitive with a large *J* (e.g., *J* > 100). Thus, in these cases, an adaptive grid search algorithm that progressively zooms in on smaller areas in the solution space is employed that effectively reduces the computational demand while maintaining reasonable accuracy. More specifically, this “zoom-in” strategy is an iterative procedure that gradually narrows the search space for the number of signaling variables until it converges to a single number. The algorithm starts with ten evenly spaced numbers (*a*_1_ < *a*_2_ < ... < *a*_10_), where *a*_1_ takes the smallest possible value and *a*_10_ takes the largest possible value. For each of these ten candidate numbers of signaling variables a CKM solution is obtained and the optimal number is selected with the Gap statistic. The algorithm then zooms in to [*a*_*i*− 1_ + 1, *a*_*i*+ 1_ − 1] (both sides included) and creates ten new evenly spaced numbers. This step is repeated until convergence.

## Simulation studies

To evaluate the performance of CKM and of the proposed model selection strategy, three simulation studies were carried out. In the first two simulation studies, we compared the performance of CKM in recovering the clusters and the status of the variables (signaling versus irrelevant) with that of SAS and of SKM. The two simulation studies differed in the amount of prior information: while both *K* (i.e., the number of clusters) and *V* (i.e., the number of irrelevant variables) were assumed to be known in simulation study 1, only the true value of *K* was provided in simulation study 2. In addition to SAS and SKM, in simulation study 2, CKM was also compared to KM. In simulation study 3, our proposed strategy that relies on the stable set of signaling variables for selecting the number of clusters and identifying the set of signaling variables was compared to the alternative – and widely applied – selection strategy that selects *K* based on the full set of variables.

All of the analyses were carried out in the statistical software R. We used our self-developed package “CKM” for the CKM algorithm, the package “stats” for the KM algorithm, and the package “sparcl” for the SKM algorithm. The SAS algorithm was available from standalone functions that were extracted from the GitHub page (see Arias-Castro & Pu, 2017). When running CKM, SAS, and SKM in Simulation 2 and 3, one hyper-parameter must be tuned for each method to select the optimal number of signaling variables. For CKM, we have elaborated the procedure to tune the cardinality constraint in the “Model selection” section. The procedure to tune the hyper-parameter for SAS is similar to that for CKM: according to Arias-Castro and Pu (2017), here too the optimal number of signaling variables is determined by maximizing the Gap statistic calculated from Eq. 12. For SKM, the tuning parameter *s*, associated with the *l*_1_ norm, should be decided for each of the simulations. *s* is tuned from a grid consisting of 200 evenly spaced values ranging from 1.001 to 10. For Simulation 1 where the number of irrelevant variables *V* is known prior to data analysis, we first determine the number of irrelevant variables *V*_0_ for each value *s*_0_ on the grid. Then, the tuning parameter *s* is selected such that its corresponding *V*_0_ equals *V*. In case multiple *V*_0_ equal *V*, the average value of their associated *s*_0_ is used. For Simulations 2 and 3 where *V* is determined during data analysis, the optimal value is selected that results in the simplest model (i.e., the model with the fewest number of signaling variables) with a Gap statistic less than 1*SE* away from the maximum. In other words, the tuning procedure for SKM follows the well-known 1*SE* rule, as proposed in Witten and Tibshirani (2010).^4^ In the above tuning process, the Gap statistic must be computed for each candidate value; here, we set the number of permutation samples to 20 for all analyses.

### Simulation study 1

In this simulation study we compared the accuracy of CKM in recovering the clusters and signaling variables with SAS and SKM; where the values of *K* and *V* were set at pre-defined values. To facilitate a systematic comparison with other studies, we adopted, as closely as possible, the data generation procedure from Witten and Tibshirani (2010) and Arias-Castro and Pu (2017). More specifically, the simulation was designed as follows: (1) the number of clusters *K* was either 3, 5, or 30; (2) the number of observations per cluster was 50; (3) the number of irrelevant variables *V* took one of the following four values: 5, 50, 250, and 1000; (4) the number of signaling variables (i.e., *J* − *V*) was 50 and (5) the distance of centroids for each variable between neighboring clusters Δ*μ* equaled one of the following four values: 0.6, 0.7, 0.8, 1. A fully crossed design was used, resulting in 3 × 1 × 4 × 1 × 4 = 48 conditions.

To generate the data, each observation was assigned to one of the *K* clusters such that all clusters were of equal size. Then, irrelevant variables were generated by drawing from the standard normal distribution. The responses on the signaling variables were sampled independently for each cluster from a normal distribution with a cluster-specific mean and a standard deviation of 1. The cluster-specific mean values were determined such that the grand mean calculated over all clusters was 0 while differences in neighboring clusters were fixed at Δ*μ*. For example, when Δ*μ* equaled 0.6, the cluster-specific mean values of the three clusters for each variable were respectively -0.6, 0, and 0.6. Obviously, a smaller Δ*μ* corresponds to closer cluster centroids, and thus results in a more difficult task to recover the clusters.

For each condition, 40 data sets were generated. Therefore, a total of 1920 data sets were generated and analyzed by CKM, SAS, and SKM. Note that, SKM was eventually dropped for the data sets generated in the conditions with 30 clusters because of its slow computation.

Following Chipman and Tibshirani (2006), Witten and Tibshirani (2010), and Arias-Castro and Pu (2017), we used classification error (CE) as the evaluation criterion of cluster recovery. By reporting CE, we hope to provide future research with a consistent point of comparison, which is particularly beneficial for studies where different methods are synchronized and (or) compared. CE indicates the similarity between the true cluster assignment *c*_*true*_ and the assignment *c*_*est*_ resulting from a particular clustering algorithm. To illustrate, we introduce the following notation: $1_{\mathbf {c}(i,i^{\prime })}$ equals 1 when observations *i* and *i*^′^ belong to the same cluster and 0 when they do not. Then, CE is defined as follows,

13 $$ \begin{array}{@{}rcl@{}} CE = \frac{{\sum}_{i>i^{\prime}}|1_{\mathbf{c_{true}}(i,i^{\prime})} - 1_{\mathbf{c_{est}}(i,i^{\prime})}|}{N(N-1)/2},\\ \text{where}~N~\text{is the total number of observations.} \end{array} $$

CE in Eq. 13 takes values between 0 and 1; CE = 0 indicates a perfect agreement between *c*_*true*_ and *c*_*est*_ while higher values indicate larger classification error and thus less agreement between these two partitions.

Furthermore, to quantify how well an algorithm retrieved the signaling variables, we computed the proportion of true signaling variables that were successfully identified by the algorithm relative to the total number of signaling variables (e.g., if 40 of the 50 signaling variables have been identified, the success rate will be 80%). Hence, a larger proportion suggests a better performance of the algorithm in detecting the signaling variables.

**Fig. 1:**
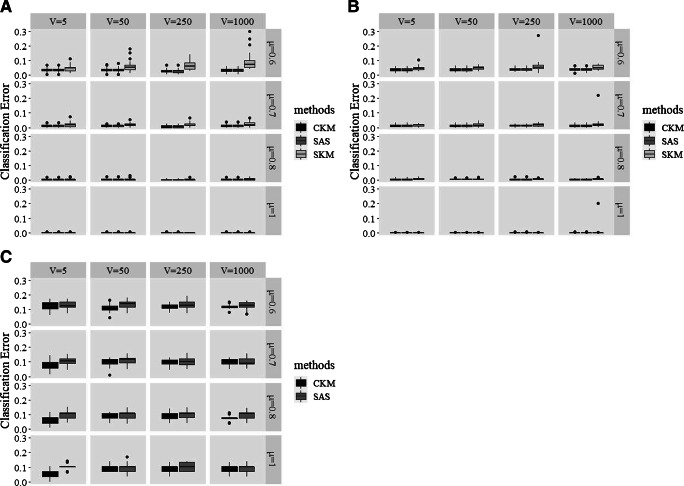
A comparison of different clustering methods for cluster recovery when both the number of clusters K and signaling variables V are given. Panel A: *K* = 3; Panel B: *K* = 5; Panel C: *K* = 30

The relative performance of CKM, SAS, and SKM in recovering the clusters are visualized in Fig. 1. Figure 1a and b shows that, when *K* equaled 3 or 5, CKM and SAS recovered the clusters equally well (for both methods, average CE = .012 when *K* = 3; average CE = .014 when *K* = 5) and both better than SKM (average CE = .025 when *K* = 3; average CE = .021 when *K* = 5). Furthermore, CKM (average CE = .092) outperformed SAS (average CE = .109) when *K* = 30 (see Fig. 1c; note that, as discussed earlier, SKM was dropped in these conditions), i.e., in the presence of a more complex cluster structure.

Next, we examined how well the three methods were able to identify the set of signaling variables. We found that the task of identifying the set of signaling variables proved to be relatively easy given the true values of both *S* and *K*: all three methods were able to identify the set of signaling variables with a success rate of at least 99%.

### Simulation study 2

Our objective in Simulation 2 was to further examine the relative performance of CKM, compared to SAS and SKM, in recovering clusters and the status of variables when only *K* was given; hence, *V* as well as the subset of signaling variables had to be determined by the algorithm. Furthermore, we have also added (standard) KM – the most commonly used algorithm that does not allow for variable selection – to the comparison and evaluated the relative performance of all four methods in terms of cluster recovery. The settings and the data generation procedure were identical to those used in Simulation 1.

In Simulation 2, again a total of 48 conditions were manipulated with 40 data sets each. This resulted in a total of 1920 data sets. We assessed the performance of the four clustering algorithms primarily based on the recovery of clusters (indicated by CE) and the number of variables identified as signaling variables. In addition, we also recorded and compared the average running time for each of the methods.

**Fig. 2:**
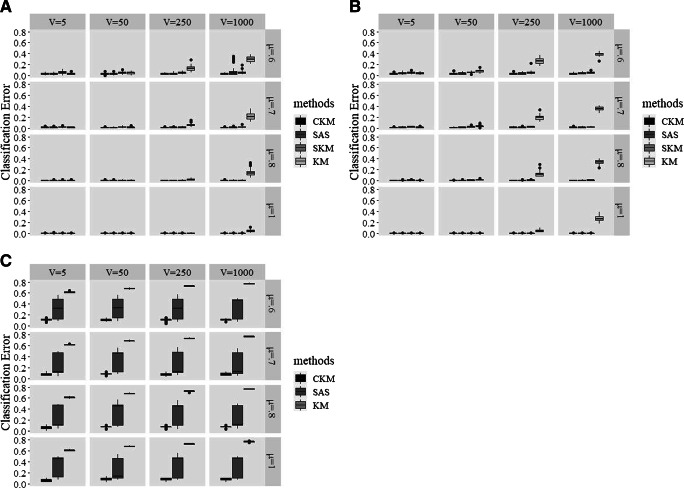
A comparison of different clustering methods for cluster recovery when only the number of clusters K is given. Panel A: *K* = 3; Panel B: *K* = 5; Panel C: *K* = 30

Figure 2a and b visualize the extent of cluster recovery by the different methods, when *K* equaled 3 and 5, respectively. Because the two subplots present a similar pattern of the relative performance of the four methods (CKM, SAS, SKM, and KM), we discuss the combined results here. Averaged over all conditions, CKM was the winner with an average CE of .013, followed by SAS (average CE = .016) and SKM (average CE = .023). KM, on average, produced cluster partitions with a CE equaling .10. With regard to the effect of Δ*μ*, the largest advantage of CKM (average CE = .035) over the other four algorithms (for SAS, average CE = .045; for SKM, average CE = .057; for KM, average CE = .16) was found when Δ*μ* = .6 (i.e., the smallest distance of centroids between neighboring clusters). We also examined how well these methods recovered clusters with respect to the different numbers of irrelevant variables (i.e., *V*). In accordance with our expectation, the three methods performing simultaneous variable selection and clustering (i.e., CKM, SAS, and SKM; for CKM, average CE = .014; for SAS, average CE = .021; for SKM, average CE = .024) recovered the clusters considerably better than KM (average CE = .26) in the presence of an exceedingly large proportion of irrelevant variables (i.e., *V* = 1000). Last, in accordance with our expectation, the performance advantage of CKM over SAS and KM in terms of cluster recovery was greatest when *K* = 30 (see Fig. 2c; for CKM, average CE = .08, for SAS, average CE = .30, for KM, average CE = .70). This again illustrates that CKM is particularly powerful to deal with complex cluster structure. When *K* = 30 and *V* = 1000, the difference in cluster recovery from the three methods is striking: the average CEs for CKM, SAS, and KM were .09, .28, and .77, respectively.

**Fig. 3:**
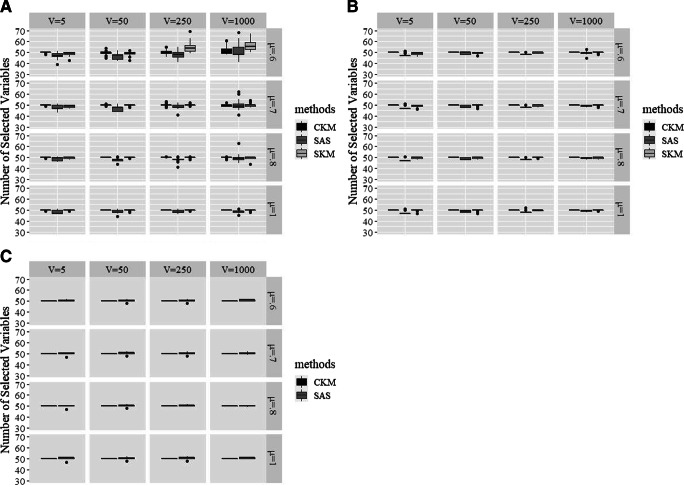
A comparison of different clustering methods for variable selection when only the number of clusters *K* is given. Panel A: *K* = 3; Panel B: *K* = 5; Panel C: *K* = 30

We further evaluated how well the algorithms identified the set of 50 signaling variables when the correct number of irrelevant variables (i.e., *V* ) was not given. Since KM is not able to explicitly single out signaling variables, the comparison only concerns CKM, SAS, and SKM – note that the true value was always 50. The results, plotted in Fig. 3, shows that CKM was the best performing method in terms of successful variable selection, since the number of variables selected by CKM was consistently close to 50, even with *V* = 1000. In contrast, with a larger number of irrelevant variables (i.e., *V* = 250 or 1000), both SAS and SKM experienced difficulty to recover the exact 50 signaling variables. Expressed in numbers, while CKM recovered the exact 50 variables in 92.7% of the cases; for SAS and SKM, this percentage of successful recoveries was only 62.9% and 30%, respectively.

Last, we examined the average execution time for each of the clustering methods (here, we only consider *K* = 3 and *K* = 5, because these are the typical scenarios behavioral researchers commonly encounter). With an average execution time of .16 s and 4.28 s, respectively, KM and SAS were the two fastest algorithms. CKM ranked third among all four methods, taking an average of 43.5 s to analyze a data set. In our opinion, its speed is acceptable for most empirical studies. SKM, with an average of 293.6 s, was a lot slower than the other three algorithms.

### Simulation study 3

Our major objective in Simulation Study 3 was to evaluate and compare different model selection procedures for deterministic clustering algorithms that perform simultaneous clustering and variable selection (e.g., CKM, SAS, and SKM). To achieve this, we examined the relative accuracy of selecting *K* with regard to (1) the set of variables used (i.e., either relying on a stable set of variables that were selected consistently across all possible numbers of clusters or the full set of variables), and (2) the selection criteria for determining the number of clusters.

A key interest in the current comparison was to compare our novel strategy that pre-selected a stable set of variables (see the previous section) with the traditional strategy that involved all variables. Our expectation was that, with a relatively large proportion of irrelevant variables, the traditional strategy considered too much noisy information and therefore resulted in less accurate selection compared to our novel strategy. Besides, we have also implemented and tested another strategy – called the local selection strategy. This strategy first selects *V* conditional upon each possible value of *K* with the *Gap*(*V* ) statistic and then selects *K* that maximizes the associated *Gap*(*V* ) statistic. However, in all conditions, this strategy consistently selected the smallest value of *K* (i.e., 2). Because of the poor performance of this strategy, we do not report its results any further in the paper study.

In the current study, we considered some of the most popular model selection criteria, namely the “KL Index” (Krzanowski & Lai, 1988), the “DIndex”(Lebart et al., 1995), and two versions of the Gap statistic (Tibshirani et al., 2001), and examined which selection criteria determined *K* with the highest accuracy. Specifically, in the current study, the following two Gap-based criteria were investigated: 1) selecting *K* that corresponded to the global maximum of the Gap statistic, called “globalGap”; and 2) choosing *K* that was associated to the first local maximal value of the Gap statistic, called “firstGap”. While the first one was proposed in Tibshirani et al., (2001), the second one was introduced in Maechler et al., (2012) in developing the well-known R package “Cluster”.

Furthermore, in the current study, to evaluate the generalizability with respect to the preferred selection strategy and selection criterion, we replicated our findings with both CKM and SAS (SKM was not involved because, as illustrated above, it was relatively slow compared to CKM and SAS).

To summarize, in Simulation Study 3, we tested the accuracy of selecting *K* with respect to three factors: (1) the selection strategy (i.e., the proposed strategy that utilizes a stable set of variables versus and a strategy that utilizes the full set of variables), (2) the selection criterion (i.e., “globalGap” v.s. “firstGap” v.s. “KL Index” v.s. “DIndex”), and (3) the clustering algorithm (i.e., CKM v.s. SAS).

A number of factors in the data generation process were systematically manipulated. These were largely identical to those of the first two simulation studies, yet, with the following exception. Namely, the varying number of clusters *K* was one of three values: 3, 5, or 15. Again, in total 3 × 4 × 4 = 48 conditions were manipulated. For each of the conditions, again 40 replicate data sets were generated, leading to a total of 1920 data sets. For each data set, *K* was selected among models with 2 up to 10 clusters clusters when *K* = 3 or *K* = 5 and among models with 11 up to 19 clusters when *K* = 15.^5^ Specifically, three model selection strategies (i.e., utilizing the stable set of variables obtained from (1) CKM, or (2) SAS, and (3) utilizing the full set of variables) combined with four model selection criteria (i.e., (1)“globalGap”, (2) “firstGap”, (3)“KL Index”, and (4) “DIndex”) were employed to analyze each of the data sets. That is, for each data set, we applied a total of 12 different ways for selecting the number of clusters *K*.

Table 1 presents the results of Simulation Study 3. Most importantly, the novel selection strategy for selecting the number of clusters that relies on the stable set of variables led to an equal or higher success rate in selecting the true number of clusters, across all criteria and conditions, and both for CKM and for SAS, in comparison with using the full set of variables. This advantage was especially pertinent in the presence of a large proportion of irrelevant variables (i.e., when *V* = 250 or *V* = 1000) where these irrelevant variables likely hampered the recovery of cluster structure and (or) in the presence of a large number of clusters (i.e., when *K* = 15). By first filtering out the irrelevant variables and only retaining the signaling variables that clearly separate the clusters, the stable set of variables offered a more defined structure for model selection, even in the presence of a large amount of clusters. In fact, the proposed model selection strategy, when coupled with the selection criteria “globalGap” or “firstGap” and the CKM or SAS algorithm, achieved a remarkable 100% recovery in all conditions examined.

**Table 1:** Percentage of correct recovery of the number of clusters for 12 different strategies to determine the number of clusters

K	V	Full set of Variables				Stable set obtained with SAS				Stable set obtained with CKM			
		gp	fp	KL	Dindex	gp	fp	KL	Dindex	gp	fp	KL	Dindex
3	5	100%	100%	87.5%	94.4%	100%	100%	91.3%	100%	100%	100%	87.5%	100%
	50	66.3 %	96.9%	67.5%	100%	100%	100%	90.6%	100%	100%	100%	88.8%	100%
3	250	32.5%	75%	6.3%	61.3%	100%	100%	99.4%	82.5%	100%	100%	83.8%	100%
	1000	6.3%	70.6%	0%	31.3%	100%	100%	99.4%	82.5%	100%	100%	83.8%	100%
5	5	58.1%	60.6%	81.9%	96.9%	100%	100%	88.1%	97.5%	100%	100%	75.6%	97.5%
	50	73.1%	95.6%	0%	19.4%	100%	100%	81.3%	93.8%	100%	100%	81.3%	93.8%
5	250	27.5%	51.9%	0%	0%	100%	100%	56.9%	80.6%	100%	100%	79.4%	91.3%
	1000	0%	0%	0%	0%	100%	100%	85.6%	89.4%	100%	100%	85.6%	90%
15	5	0%	0%	19.4%	0%	100%	100%	13.1%	48.1%	100%	100%	0.6%	7.5%
	50	0%	0%	40.6%	40.6%	100%	100%	12.5%	26.3%	100%	100%	0%	6.3%
15	250	0%	0%	0%	0%	100%	100%	12.5%	26.3%	100%	100%	0%	6.3%
	1000	0%	0%	0%	0%	100%	100%	16.9%	10.6%	100%	100%	0%	5%

*Note:* Stable set refers to the proposed approach where only the stable set of signaling variables are used for selecting the number of clusters; full set refers to the conventional approach where all variables are used. gp = “globalGap”, fp = “firstGap” (see the text for detailed explanation of the two statistics)

### Summary of the simulation studies

In three simulation studies we evaluated (1) the relative performance of CKM with respect to SAS, SKM and KM in cluster recovery and the selection of signaling variables with (“Simulation study 1”) and without (Simulation study 2) a pre-determined number of irrelevant variables, and (2) the accuracy of selecting the number of clusters for all possible combinations of three variable selection strategies and four indices for determining the number of clusters. Our main findings were as follows: first, compared to the three competing methods – namely SAS, SKM and KM, CKM was the winner in terms of cluster recovery across various conditions, with or without model selection. Second, in comparison to the other methods that are also capable of identifying signaling variables (i.e., SAS and SKM), CKM was the most accurate one when the number of irrelevant variables was unknown and the cluster structure was complex. Third, SAS enjoyed the shortest execution time in comparison to CKM and SKM. Fourth, we found that, across all conditions, the proposed model selection strategy that utilizes the stable set of variables resulted in a better accuracy in selecting the number of clusters compared to the traditional strategy that utilizes the full set of variables. Finally, the best model selection procedure consisted of the combination of the proposed model selection strategy that relies on the stable set of signaling variables and the index “globalGap” or “firstGap”. In our simulation setup, this procedure led to perfect performance of CKM and SAS.

## Application

Here, we demonstrate the usefulness of CKM in analyzing an empirical data set. We consider gene expression data of 13 autistic subjects and 14 healthy subjects that are publicly available from the gene expression omnibus (GEO) with accession number GSE7329.^6^ For each subject, the transcription rates of 43,893 probes were analyzed. Therefore, the data used in our analyses includes a total of 27 rows (subjects) and 43893 columns (variables). According to Nishimura et al., (2007), only a small number of probes are associated to autism – in their research, the authors selected a total of 293 probes for which the analysis of variance (ANOVA) tests resulted in a false discovery rate below a threshold of 5%.

**Fig. 4:**
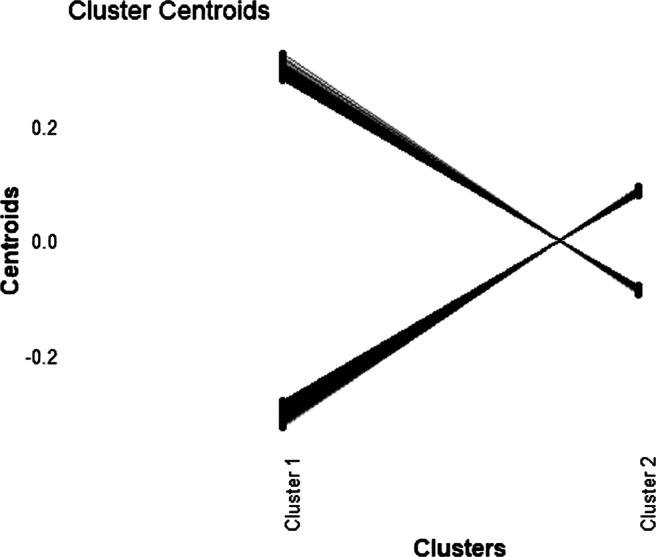
The cluster-specific centroids of the probes that were involved in key disease-related pathways

Before the analysis, we have pre-processed the data set such that each of the variables was mean-centered and scaled to unit sum-of-squares. Our first set of analyses was based on the full set of 43,893 variables. More specifically, CKM, SAS and KM were applied to the entire data set with *K* specified at 2 - to represent the autistic group and the control group. We did not try out a larger number of clusters considering the very small sample size. The three methods (i.e., CKM, KM, and SAS) all resulted in the same cluster partition: the first cluster contained the subjects with the indices 5, 6, 9, 15, 16, and 27 while the second cluster contained the remaining 21 subjects. Note that this partition was different from the assumed partition separating the patients (with the indices 1–14) and the control group (with the indices 15–27). The disparity between the known partition and the obtained partition is probably due to the presence of other biological mechanisms. To support this hypothesis, we further inspected the probes selected by the algorithms. While CKM selected a total of 958 probes, SAS selected 1238 probes. We used the free functional annotation tool DAVID (Bioinformatics Resources Version 6.8; Huang et al., 2007) to explore if the set of signaling variables identified by CKM indeed corresponds to any meaningful biological processes. The annotation picked up three groups of genes that were related to pathways that play an important role in three different types of disease: 20 genes were involved in the pathway of Parkinson’s Disease; 22 in the pathway of Alzheimer’s disease; 22 in the pathway of Huntington’s disease. Given that the autistic subjects had a single gene Mendelian disorder (either a 15q11-q13 duplication or a fragile X mutation) and that the control subjects were composed of non-autistic siblings, it is not unlikely that a grouping structure is present in which autistic and control subjects are mixed. Figure 4 offers a visualization of cluster-specific centroids (after pre-processing) of all 958 signaling probes, with the line linking the two centroids of the same variable for the two clusters. Clearly, the two clusters showed distinctive response patterns: while a group of variables were associated with positive values in Cluster 1 and negative values in Cluster 2, the other group of variables showed a directly opposite pattern. We stress that the current analysis should only be regarded as an exploratory analysis and further studies are needed to confirm the relevance of the two obtained clusters and their distinct genetic profiles.

We then conducted a second set of analyses where we used a subset of variables from the original data set. The subset consisted of two types of variables: the 293 signaling variables that have a significant difference in means between the autistic group and the control group and 1707 variables that were chosen randomly from the remaining variables (the new data set thereby involved a total of 2000 variables). To determine the signaling variables, we conducted a total of 43,893 sets of linear regressions that regressed the transcription rate of each probe on the known partition of subjects with or without autism, and chose the 293 variables with the highest values of regression coefficients. Compared to the previous cluster analysis, we were more certain that the primary factor that divided all subjects was whether they were autistic or not. Consequently, we were able to evaluate the empirical performance of the clustering methods by examining to what extent a method successfully recovered the cluster partition and the set of signaling variables. CKM completed the task perfectly as it identified the exact 293 variables that were pre-defined as signaling variables. SAS also identified all of the 293 pre-defined signaling variables; however, in addition to this, it also erroneously picked 23 of the pre-defined irrelevant variables as if they were signaling variables.

To summarize, although the pre-existing groups were not recovered when the full data set was used, probably because of the existence of other biological processes that divided the subjects, the cluster structure was successfully recovered by CKM in a chosen subset of the data (with a total of 2000 probes). In terms of the accuracy of variable selection, in accordance with our findings in the simulation studies, CKM clearly outperformed SAS as it recovered the subset of signaling variables perfectly.

## General discussion

Although behavioral sciences have a long tradition of operating in a “theory-driven way” and hence typically work with a small number of carefully selected and designed variables, they are now opening up its door to an interdisciplinary, data-rich approach where data sets involving many variables are increasingly common (Gil de Zuniga and Diehl, 2017). The growing availability of these data sets and the adoption of a data-driven approach could largely contribute to exploratory research (Fan et al., 2014; Yuan et al., 2021). In the context of cluster analysis, for example, the application of data-driven approaches to high-dimensional data could potentially lead to the discovery of novel subgroups that are not detectable from a traditional examination (Yuan et al., 2019). Yet, a unique challenge of this approach pertains to retaining only crucial variables that truly separate the clusters and filtering out irrelevant variables. Successfully identifying these signaling variables is beneficial to the recovery as well as the interpretation of the underlying clusters.

To address this challenge and facilitate data exploration with high-dimensional data sets, several methods – for example, Sparse *k*-means (SKM) and Sparse Alternate Sum (SAS) – have been proposed that perform simultaneous clustering and variable selection. In the current study, we contributed to this line of research in two important ways. First, we presented a novel method, called Cardinality *K*-means, or CKM, that exploits the connection between PCA and KM to obtain, in a computationally efficient way, good starting values for a K-means (KM) procedure with variable selection. Our specific contribution is to introduce a special variant of the sparse principal component analysis (SPCA) with a cardinality constraint on the number of variables. As a result, CKM is a method that is similar to SAS, but with a much better initiation of the parameter values. Through extensive simulations that included a number of important factors (e.g., the number of clusters, the proportion of irrelevant variables, and the distance between the centroids of adjacent clusters), we confirmed that CKM outperformed the other clustering methods (i.e., SAS, SKM, and KM) in terms of cluster recovery, especially in the presence of a large number of irrelevant variables. Furthermore, among the three methods with simultaneous variable selection (i.e., SAS, SKM, and CKM), CKM enjoyed the highest success rate in the identification of signaling variables. Compared to its predecessors SKM and SAS, CKM not only recovers clusters better, but also offers a more structured and flexible approach to simultaneous clustering and variable selection. CKM uses the cardinality constraint, which offers at least the following two advantages over the *l*_1_ penalty used in SKM. First, the application of the cardinality constraint (but not the *l*_1_ penalty) allows users to have exact control over the number of signaling variables (Guerra-Urzola et al., 2021). This option is particularly helpful when a pre-specified number of signaling variables is desired in certain applications. Second, the *l*_1_ penalty has long been criticized as suboptimal when the primary task is variable selection, and in such tasks, regression analysis with an *l*_1_ penalty under-performed that with a cardinality constraint (e.g., Bertsimas et al., 2016). Moreover, thanks to the structured SPCA step, CKM can be easily extended to account for different types of analyses, which is not possible with SAS. For example, a researcher may want to find a specific structure of four clusters in which irrelevant variables only pertain to two clusters, while for the other two clusters, all variables are considered signaling variables. To accommodate this structure, in the first step where SPCA is performed, the cardinality constraint can be imposed for only two columns of the loading matrix. Furthermore, in the second step where the model parameters of CKM are iteratively updated, the loss function can be adjusted to reflect this assumption.

Another important contribution to the literature is that we proposed a novel model selection strategy to determine the number of clusters *K*. The proposed strategy adopts a three-step procedure that first applies a simultaneous clustering and variable selection algorithm (e.g., CKM, SAS or SKM) to identify the most stable set of variables, i.e., those consistently identified as signaling variables given any of the considered values of *K*, and then relies on this subset of variables to select the optimal value of *K*. Through simulation study 3, the proposed strategy – using either SAS or CKM to extract the stable set of variables – recovered *K* more accurately than the traditional strategy that selects *K* based on the full set of variables. Furthermore, we also found that, among the four evaluated model selection criteria (i.e., “globalGap”, “firstGap”, “KL Index”, and “DIndex”), the two criteria developed from the Gap statistic (Tibshirani et al., 2001) recovered *K* with the highest accuracy. Overall, our study indicated that the preferred procedure of selecting *K* consists of two steps: (1) apply either CKM or SAS for each possible value of *K* and identify a stable set of variables that are consistently estimated as signaling variables; (2) determine *K* based on the stable set of variables with either “globalGap” or “firstGap”.

To conclude, We strongly advocate the use of a simultaneous variable selection and clustering approach (e.g., CKM, SAS, and SKM) when the data contains a large number of variables and (or) it is desirable to pick up a subset of the most important variables – e.g., for the purpose of data exploration. When choosing between CKM, SAS, and SKM, according to the aforementioned results, we recommend the application of CKM when the primary objective is to recover the clusters and signaling variables as much as possible. When speed is important (e.g., in dealing with streaming data), however, SAS is the most desirable method. Last, the selection of the number of clusters is preferably based on a stable set of signaling variables that partial out irrelevant variables as much as possible.

We see several interesting future directions for CKM. First, in applications, the underlying cluster structure may be more complex than those generated in the simulations. Here, we discuss two scenarios that researchers may encounter and briefly elaborate how CKM can be used in both scenarios. Consider a hypothetical data set with 200 variables and six clusters. In the first scenario, there is only one way of partitioning subjects and different subsets of clusters are separated by different subsets of variables (e.g., the first 50 variables are relevant to Clusters 1–3 but not to Cluster 4–6, the last 50 are relevant to Cluster 4–6 but not to Cluster 1–3, and the other 100 variables are completely irrelevant to all clusters). When dealing with this data set, we expect CKM to successfully recover the six clusters and select variables 1–50 and 151–200 as signaling variables. After retrieving the full set of signaling variables, users can then inspect the centroids of these variables for the six clusters to discover which subsets of variables are relevant to which subsets of clusters. In the second scenario, completely different partitions (i.e., with hardly any agreement between the two partitions) of the subjects pertain to different subsets of variables. In our hypothetical data set with 200 variables, all subjects may be partitioned to six clusters in two different ways: the first partition is driven by the first 50 variables, the second is driven by the last 50 variables, and the remaining 100 variables are once again irrelevant. To account for this scenario, users of CKM can follow an iterative procedure: after each step of identifying clusters and selecting signaling variables, the algorithm proceeds to apply CKM to the designated irrelevant variables. To prevent overfitting (i.e., finding clusters and associated signaling variables that are caused by noise only), after each step, theoretical knowledge can be used to confirm the clusters while resampling methods – e.g., bootstrapping and permutation test – can be applied to examine the stability of these clusters. We encourage future research to systematically examine the performance of these strategies in various applications. Second, future studies could investigate how different types of initialization affect the results of CKM. A notable limitation of the current simulation study is that, when initializing the alternating procedure for estimating CKM solutions (i.e., Step 2), we utilized only one rational start, estimated from a procedure inspired by USLPCA, yet we did not consider a multi-start procedure that employs multiple random starts. However, we would also like to point out that, according to Xu et al., (2015), a PCA-guided rational start likely yields comparable performance as a multi-start procedure when estimating KM results. Third, currently, CKM is only able to deal with continuous data with no missing responses. In future research, different imputation methods could be evaluated and compared, resulting in a preferred pre-processing scheme for a CKM analysis. Moreover, an extension of CKM can be developed to tackle mixed types of data (i.e., a combination of nominal, ordinal, and continuous variables).

## Appendix A: An alternative formulation of KM

In this section, our goal is to illustrate that the objective function of a KM analysis could be re-formulated as **a****r****g****m****a***x*_**H**_(*T**r***H**^′^**X****X**^′^**H**), subject to **H**^′^**H** = **I**_*k*_ and an orthogonality constraint imposed on **H**.

First, we acknowledge that Eq. 1 could be re-written in

14 $$ \begin{array}{@{}rcl@{}} \mathbf{argmin_{C}}{\sum}_{k=1}^{K}{\sum}_{i \in C^{-1}(k)}||\mathbf{x}_{i} - \mathbf{m}_{k}||_{2}^{2} &=& \mathbf{argmin_{C}}({\sum}_{i=1}^{N}||\mathbf{x_{i}}||_{2}^{2}\\ &&- {\sum}_{k=1}^{K}\mathbf{m}_{k} {\sum}_{i \in C^{-1}(k)}(2\mathbf{x}_{i} - \mathbf{m}_{k})^{\prime}) \\ &=& \mathbf{argmin_{C}}({\sum}_{i=1}^{N}||\mathbf{x}_{i}||_{2}^{2}\\ &&- {\sum}_{k=1}^{K}n_{k}\mathbf{m}_{k}\mathbf{m}_{k^{\prime}}). \end{array} $$

Per Eq. 3, **m**_*k*_ in Eq. 14 could be further replaced by 1*n*_*k*_**h***k*′**X**, resulting in

15 $$ \begin{array}{@{}rcl@{}} \mathbf{argmin_{C}}({\sum}_{i=1}^{N}||\mathbf{x}_{i}||_{2}^{2} - {\sum}_{k=1}^{K}n_{k}\mathbf{m}_{k}\mathbf{m}_{k^{\prime}}) &=& \mathbf{argmin_{\mathbf{H}}}({\sum}_{i=1}^{N}{\sum}_{j=1}^{J}x_{ij}^{2}\\ &&- {\sum}_{k=1}^{K} \mathbf{h}_{k}^{\prime}\mathbf{X}\mathbf{X}^{\prime}\mathbf{h}_{k})\\ &=& \mathbf{argmin_{\mathbf{H}}}(||\mathbf{X}||_{2}^{2}\\ &&- Tr\mathbf{H}^{\prime}\mathbf{X}\mathbf{X}^{\prime}\mathbf{H}). \end{array} $$

The last part of the equation holds because of the orthogonality of **H**.

## Appendix B: The equivalence of the two optimization formulations that concern KM with irrelevant variables

In the current section, we discuss the equivalence between Eqs. 7 and 10.

We apply the equivalence of Eqs. 6 and 5 in Eq. 9 and obtain

$$ \begin{array}{@{}rcl@{}} \mathbf{argmax}_{\hat{\mathbf{H}}, \mathbf{g}} Tr\hat{\mathbf{H}}^{\prime}\mathbf{X_{-g}}\mathbf{X_{-g}}^{\prime}\hat{\mathbf{H}} & \Leftrightarrow\mathbf{argmin}_{\hat{\mathbf{H}},\mathbf{P,\mathbf{g}}}||\mathbf{X_{-g}} - \hat{\mathbf{H}}\mathbf{P}_{-g}^{\prime}||_{2}^{2}. \end{array} $$

Therefore, Eq. 7 could be reformulated in

$$ \begin{array}{@{}rcl@{}} &&{\kern-.5pc}\mathbf{argmin_{\mathbf{g}}}||\mathbf{X_{g}}||_{2}^{2} + \mathbf{argmin}_{\hat{\mathbf{H}},\mathbf{P,\mathbf{g}}}||\mathbf{X_{-g}} - \hat{\mathbf{H}}\mathbf{P}_{-g}^{\prime}||_{2}^{2} \Leftrightarrow\mathbf{argmin}_{\hat{\mathbf{H}},\mathbf{P}}||\mathbf{X} - \hat{\mathbf{H}}\mathbf{P}^{\prime}||_{2}^{2}\\ &&{\kern-.5pc}\text{with \(\mathbf{P}\) containing \(V\) roes of zero entries.} \end{array} $$

## Appendix C: Proof for procedures to update **Ĥ** and **P**

In the current section, we provide detail derivations to support Algorithm 1. We first show the optimization problem **a****r****g****m****i****n**_**Ĥ**_||**X** −**Ĥ****P**^′^|| 22 subject to **Ĥ**^′^**Ĥ** = **I** has the solution

$$ \hat{\mathbf{H}} = \mathbf{U}\mathbf{V}^{\prime}$$

where **U** and **V** are obtained from the SVD of **X****P**.

We rewrite the optimization in

$$ \begin{array}{@{}rcl@{}} h(\hat{\mathbf{H}}) &=& ||\mathbf{X} - \hat{\mathbf{H}}\mathbf{P}^{\prime}||_{2}^{2} \\ &=& tr\mathbf{P}\hat{\mathbf{H}}^{\prime}\hat{\mathbf{H}}\mathbf{P}^{\prime} + tr\mathbf{X}^{\prime}\mathbf{X} - 2tr\mathbf{X}\mathbf{P}\hat{\mathbf{H}}^{\prime} \\ &=& tr\mathbf{P}\mathbf{P}^{\prime} + \mathbf{X}^{\prime}\mathbf{X} - 2tr\hat{\mathbf{H}}^{\prime}\mathbf{X}\mathbf{P}.\\ \end{array} $$

Therefore, the minimization problem is equivalent to the maximization problem of *t**r***Ĥ**^′^**X****P** subject to **Ĥ**^′^**Ĥ** = **I**. Such a maximization problem can be addressed with the Kristof theorem (for a detailed description and proof of the Kristof theorem, please refer to ten Berge 1993). More specifically, we realize *t**r***Ĥ**^′^**X****P** could be rephrased in

$$ \begin{array}{@{}rcl@{}} tr\hat{\mathbf{H}}^{\prime}\mathbf{X}\mathbf{P} &=& tr\hat{\mathbf{H}}^{\prime}\mathbf{U}\mathbf{D}\mathbf{V}^{\prime} \\ &=& tr\mathbf{V}^{\prime}\hat{\mathbf{H}}^{\prime}\mathbf{U}\mathbf{D} \\ &=& tr\mathbf{G}\mathbf{D}, \end{array} $$

where **X****P** = **U****D****V**^′^ represents the SVD of **X****P**.

Since **G** = **V**^′^**Ĥ**^′^**U** and all of **V**, **Ĥ**, **U** are sub-orthonormal matrices (i.e., they can be completed to orthonormal matrices), **G** is also a sub-orthonormal matrix. Therefore, according to the Kristof Theorem, *t**r***G****D** ≤ *t**r***D**, and the maxima is reached when *V*^′^**Ĥ****U**^′^ = **I**. Given the orthonormality of both **U** and **V**, **Ĥ** = **U****V**^′^.

Now consider the optimization problem **a****r****g****m****i***n*_**P**_||**X** −**Ĥ****P**^′^|| 22 subject to the constraint that *V* rows in loading matrix **P** are exact zeros. The solution of **P** is obtained in two steps: (1) calculate *P*_0_ = **X**^′^**Ĥ** and (2) impose zeros on the *V* rows of *P*_0_ whose sum-of-squares are smallest.

We re-write the optimization problem in

$$ \begin{array}{@{}rcl@{}} h(\mathbf{P}) &=& ||\mathbf{X} - \hat{\mathbf{H}}\mathbf{P}^{\prime}||_{2}^{2}\\ &=& tr\mathbf{X}^{\prime}\mathbf{X} + tr\mathbf{P}\hat{\mathbf{H}}^{\prime}\hat{\mathbf{H}}\mathbf{P}^{\prime} - 2tr\mathbf{P}\hat{\mathbf{H}}^{\prime}\mathbf{X}\\ &=& Const. + tr\mathbf{P}^{\prime}\mathbf{P} - 2tr\mathbf{P}\mathbf{W}\\ &=& Const. + {\sum}_{j=1}^{J}{\sum}_{k=1}^{K}p_{jk} - 2{\sum}_{j=1}^{J}{\sum}_{k=1}^{K}p_{jk}w_{jk}\\ &=& {\sum}_{j=1}^{J}{\sum}_{k=1}^{K}(p_{jk} - w_{jk})^{2} - {\sum}_{j=1}^{J}{\sum}_{k=1}^{K}w_{jk}^{2}\\ &=& {\sum}_{j=1}^{J}({\sum}_{k=1}^{K}(p_{jk} - w_{jk})^{2}) + Const. - {\sum}_{j=1}^{J}{\sum}_{k=1}^{K}w_{jk}^{2}, \end{array} $$

where *C**o**n**s**t*. = ||**X**||22 is a constant and **W** = **X**^′^**Ĥ**. Note that ∑ *j*= 1*J*∑ *k*= 1*K**w**j**k*2 is also a constant. Hereby, we derive the solution to **P**.

## Footnotes

1 We have also tested direct use of **c** and **g** as the partition of the samples and set of irrelevant variables, respectively. This procedure gave unsatisfactory results.

2 They also reported other suggestions for initialization; yet these different of initialization all lead to similar results.

3 Alternatively, this optimal value can be found automatically by identifying the global or local maximum of scree rations (please see an illustration in De Roover et al., 2012; also see a detailed description of the automation procedure in the CKM tutorial available on Github).

4 Note that, for SKM, we also tried in a small-scale simulation to determine the hyper parameter by maximizing the Gap statistic; however, the results of the simulation were more in favor of the selection with the 1SE rule.

5 While the range for selecting *K* was limited by the scope of the simulations, we encourage applied researchers to consider a wide range of candidate values.

6 The full data set as well as the associated material could be extracted from the following address: https://www.ncbi.nlm.nih.gov/geo/query/acc.cgi?acc=GSE7329. While the original data set contained a total of 30 subjects, we were informed that three of the subjects (with series number GSM176615, GSM176589 and GSM176586) were not correctly stored in the data set and were therefore excluded from the current analysis

## References

[CR1] Adachi K, Trendafilov NT (2016). Sparse principal component analysis subject to prespecified cardinality of loadings. Computational Statistics.

[CR2] Arias-Castro E, Pu X (2017). A simple approach to sparse clustering. Computational Statistics & Data Analysis.

[CR3] Arvey RD, Li WD, Wang N (2016). Genetics and organizational behavior. Annual Review of Organizational Psychology and Organizational Behavior.

[CR4] Bertsimas D, King A, Mazumder R (2016). Best subset selection via a modern optimization lens. The Annals of Statistics.

[CR5] Bouveyron C, Brunet-Saumard C (2014). Model-based clustering of high-dimensional data: A review. Computational Statistics & Data Analysis.

[CR6] Bouveyron, C., Celeux, G., Murphy, T.B., & Raftery, A.E. (2019). *Model-based clustering and classification for data science: With applications in R* (Vol 50). Cambridge University Press.

[CR7] Brudvig S, Brusco MJ, Cradit JD (2019). Joint selection of variables and clusters: recovering the underlying structure of marketing data. Journal of Marketing Analytics.

[CR8] Brusco MJ, Cradit JD (2001). A variable-selection heuristic for k-means clustering. Psychometrika.

[CR9] Bzdok D, Meyer-Lindenberg A (2018). Machine learning for precision psychiatry: Opportunities and challenges. Biological Psychiatry: Cognitive Neuroscience and Neuroimaging.

[CR10] Chi W, Li WD, Wang N, Song Z (2016). Can genes play a role in explaining frequent job changes? An examination of gene-environment interaction from human capital theory. Journal of Applied Psychology.

[CR11] Chipman H, Tibshirani R (2006). Hybrid hierarchical clustering with applications to microarray data. Biostatistics.

[CR12] Davis C, Zai CC, Adams N, Bonder R, Kennedy JL (2019). Oxytocin and its association with reward-based personality traits: A multilocus genetic profile (mlgp) approach. Personality and Individual Differences.

[CR13] De Roover K, Ceulemans E, Timmerman ME, Vansteelandt K, Stouten J, Onghena P (2012). Clusterwise simultaneous component analysis for analyzing structural differences in multivariate multiblock data. Psychological methods.

[CR14] Ding, C., & He, X. (2004). K-means clustering via principal component analysis. In *Proceedings of the twenty-first international conference on machine learning* (p. 29).

[CR15] Fan J, Han F, Liu H (2014). Challenges of big data analysis. National Science Review.

[CR16] Feldman R, Monakhov M, Pratt M, Ebstein RP (2016). Oxytocin pathway genes: Evolutionary ancient system impacting on human affiliation, sociality, and psychopathology. Biological Psychiatry.

[CR17] Fowlkes EB, Mallows CL (1983). A method for comparing two hierarchical clusterings. Journal of the American Statistical Association.

[CR18] Friedman JH, Meulman JJ (2004). Clustering objects on subsets of attributes (with discussion). Journal of the Royal Statistical Society: Series B (Statistical Methodology).

[CR19] Gil de Zuniga H, Diehl T (2017). Citizenship, social media, and big data: Current and future research in the social sciences. Social Science Computer Review.

[CR20] Groeneveld PW, Rumsfeld JS (2016). Can big data fulfill its promise?. Circulation: Cardiovascular Quality and Outcomes.

[CR21] Guerra-Urzola, R., Van Deun, K., Vera, J.C., & Sijtsma, K. (2021). A guide for sparse pca: Model comparison and applications. *Psychometrika*, 1–27. 10.1007/s11336-021-09773-2 PMC8636462 34185214

[CR22] Huang DW, Sherman BT, Tan Q, Kir J, Liu D, Bryant D (2007). David bioinformatics resources: Expanded annotation database and novel algorithms to better extract biology from large gene lists. Nucleic Acids Research.

[CR23] Joel S, Eastwick PW, Finkel EJ (2017). Is romantic desire predictable? Machine learning applied to initial romantic attraction. Psychological Science.

[CR24] Krzanowski, W.J., & Lai, Y. (1988). A criterion for determining the number of groups in a data set using sum-of-squares clustering. *Biometrics*, 23–34.

[CR25] Lebart, L., Morineau, A., & Piron, M. (1995). *Statistique exploratoire multidimensionnelle* (Vol. 3). Dunod Paris.

[CR26] Maechler M, Rousseeuw P, Struyf A, Hubert M, Hornik K (2012). Cluster: Cluster analysis basics and extensions. R Package Version.

[CR27] Mothi SS, Sudarshan M, Tandon N, Tamminga C, Pearlson G, Sweeney J, Keshavan MS (2019). Machine learning improved classification of psychoses using clinical and biological stratification: Update from the bipolar-schizophrenia network for intermediate phenotypes (b-snip). Schizophrenia Research.

[CR28] Nishimura Y, Martin CL, Vazquez-Lopez A, Spence SJ, Alvarez-Retuerto AI, Sigman M (2007). Genome-wide expression profiling of lymphoblastoid cell lines distinguishes different forms of autism and reveals shared pathways. Human Molecular Genetics.

[CR29] Park G, Schwartz HA, Eichstaedt JC, Kern ML, Kosinski M, Stillwell DJ, Seligman ME (2015). Automatic personality assessment through social media language. Journal of Personality and Social Psychology.

[CR30] Raftery AE, Dean N (2006). Variable selection for model-based clustering. Journal of the American Statistical Association.

[CR31] Shen H, Huang JZ (2008). Sparse principal component analysis via regularized low rank matrix approximation. Journal of Multivariate Analysis.

[CR32] Steinley D (2006). K-means clustering: A half-century synthesis. British Journal of Mathematical and Statistical Psychology.

[CR33] Steinley D, Brusco MJ (2008). A new variable weighting and selection procedure for k-means cluster analysis. Multivariate Behavioral Research.

[CR34] Steinley D, Brusco MJ (2008). Selection of variables in cluster analysis: An empirical comparison of eight procedures. Psychometrika.

[CR35] Steinley D, Brusco MJ (2011). Evaluating mixture modeling for clustering: Recommendations and cautions. Psychological Methods.

[CR36] Sun D, van Erp TG, Thompson PM, Bearden CE, Daley M, Kushan L, Cannon TD (2009). Elucidating a magnetic resonance imaging-based neuroanatomic biomarker for psychosis: classification analysis using probabilistic brain atlas and machine learning algorithms. Biological Psychiatry.

[CR37] ten Berge JM (1993). Least squares optimization in multivariate analysis.

[CR38] Tibshirani R, Walther G, Hastie T (2001). Estimating the number of clusters in a data set via the Gap statistic. Journal of the Royal Statistical Society: Series B (Statistical Methodology).

[CR39] Tseng GC (2007). Penalized and weighted k-means for clustering with scattered objects and prior information in high-throughput biological data. Bioinformatics.

[CR40] Waldherr A, Maier D, Miltner P, Günther E (2017). Big data, big noise: The challenge of finding issue networks on the web. Social Science Computer Review.

[CR41] Waldman DA, Wang D, Fenters V (2019). The added value of neuroscience methods in organizational research. Organizational Research Methods.

[CR42] Wang J (2010). Consistent selection of the number of clusters via crossvalidation. Biometrika.

[CR43] Witten DM, Tibshirani R (2010). A framework for feature selection in clustering. Journal of the American Statistical Association.

[CR44] Xu Q, Ding C, Liu J, Luo B (2015). Pca-guided search for k-means. Pattern Recognition Letters.

[CR45] Yamashita N, Adachi K (2020). A modified k-means clustering procedure for obtaining a cardinality-constrained centroid matrix. Journal of Classification.

[CR46] Yarkoni T, Westfall J (2017). Choosing prediction over explanation in psychology: Lessons from machine learning. Perspectives on Psychological Science.

[CR47] Yuan, S., De Roover, K., Dufner, M., Denissen, J.J., & Van Deun, K. (2019). Revealing subgroups that differ in common and distinctive variation in multi-block data: Clusterwise sparse simultaneous component analysis. Social Science Computer Review, 0894439319888449.

[CR48] Yuan, S., Kroon, B., & Kramer, A (2021). Building prediction models with grouped data: A case study on the prediction of turnover intention. Human Resource Management Journal.

